# Phenolic Contents and Antioxidant Activity of *Citrullus colocynthis* Fruits, Growing in the Hot Arid Desert of the UAE, Influenced by the Fruit Parts, Accessions, and Seasons of Fruit Collection

**DOI:** 10.3390/antiox11040656

**Published:** 2022-03-29

**Authors:** Shaimaa Al-Nablsi, Ali El-Keblawy, Muna A. Ali, Kareem A. Mosa, Alshaimaa M. Hamoda, Abdallah Shanableh, Ahmed M. Almehdi, Sameh S. M. Soliman

**Affiliations:** 1 Department of Applied Biology, College of Sciences, University of Sharjah, Sharjah P.O. Box 27272, United Arab Emirates; u17105705@sharjah.ac.ae (S.A.-N.); u17105693@sharjah.ac.ae (M.A.A.); kmosa@sharjah.ac.ae (K.A.M.); 2 Research Institute of Science and Engineering (RISE), University of Sharjah, Sharjah P.O. Box 27272, United Arab Emirates; shanableh@sharjah.ac.ae; 3 Department of Biology, Faculty of Science, Al-Arish University, Al-Arish 45511, Egypt; 4 College of Medicine, University of Sharjah, Sharjah P.O. Box 27272, United Arab Emirates; u20104733@sharjah.ac.ae; 5 Department of Biotechnology, Faculty of Agriculture, Al-Azhar University, Cairo 11751, Egypt; 6 Research Institute for Medical and Health Sciences, University of Sharjah, Sharjah P.O. Box 27272, United Arab Emirates; 7 Department of Pharmacognosy, Faculty of Pharmacy, Assiut University, Assiut 71515, Egypt; 8 Department of Chemistry, College of Sciences, University of Sharjah, Sharjah P.O. Box 27272, United Arab Emirates; 9 Department of Medicinal Chemistry, College of Pharmacy, University of Sharjah, Sharjah P.O. Box 27272, United Arab Emirates

**Keywords:** *Citrullus colocynthis*, season, fruit parts, total phenolic contents, antioxidant activity, metabolomics

## Abstract

*Citrullus colocynthis* (Cucurbitaceae) is an important medicinal plant traditionally used in the United Arab Emirates (UAE). In a recent study, it has been reported that different individuals of the same population of *C. colocynthis*, growing in the hot arid desert of the UAE, exhibited variations in their fruit size, color, and stripe pattern. In addition, these plants differed genetically, and their seeds showed variation in size, color, and germination behavior (hereinafter, these individuals are referred to as accessions). In the present study, the total phenolic content (TPC) and antioxidant activity of different fruit parts (rinds, pulps, and seeds) of three different accessions with significant genetic variations, from a single *C. colocynthis* population, were assessed in response to different seasonal environments. Green fruits were collected in summer and winter from three accessions growing in the botanic garden of the University of Sharjah, UAE. Methanolic extracts from different fruit parts were prepared. The TPC was qualitatively determined by a Folin–Ciocalteu assay, while the antioxidant capacity was analyzed using the 2,2-diphenyl-1-picryl-hydrazyl-hydrate (DPPH) radical scavenging ability. The metabolic profiling of the antioxidant metabolites was determined using a gas chromatograph coupled to mass spectrometry (GC–MS), associated with a literature search. The results showed that the TPC and the DPPH free radical scavenging activity varied between seasons, accessions, and fruit parts. The highest phenolics were in rinds, but the highest antioxidant activities were in seeds during the summer, reflecting the role of these compounds in protecting the developed seeds from harsh environmental conditions. The metabolomic analysis revealed the presence of 28 metabolites with significant antioxidant activities relevant to fruit parts and season. Collectively, the formation of phenolics and antioxidant activity in different fruit parts is environmentally and genetically dependent.

## 1. Introduction

The Cucurbitaceae, or the gourd family, is generally drought-tolerant and frost-sensitive [1]. This family consists of approximately 965 plant species; the most well-known members of this family that are important to humans include the cucumber, the pumpkin, the squash, the watermelon, and the bitter apple (*Citrullus colocynthis*) [2]. Genetic diversity is a very common feature in the food plants of the Cucurbitaceae [3]. *Citrullus colocynthis* (L.) Schrad is a valuable cucurbit plant widely distributed in the Arabian Peninsula, India, Africa, and other tropical regions of the world [4]. This plant shows several medicinal values such as antidiabetic, antimicrobial, anticancer, and anti-inflammatory activities [5]. In the UAE, *C. colocynthis* is one of the most popular folk medicines [6].

Different environmental conditions prevailing during the plants’ growth can affect the composition and biological activity of the plant [7]. It has been reported that samples of certain plant species collected at different times of the year showed significant differences in their chemical constituents and, consequently, different pharmacological properties [8,9,10]. For example, the concentration of active compounds was higher in plants collected in summer in some plants like *Rosmarinus officinalis* [11], *Salvia fruticosa* [12], but higher in other plants harvested in winter, such as *Leucosidea sericea* [13] and *Origanum majorana* [14]. So far, no study has assessed the seasonal variation of the phenolic contents and antioxidant activity of different parts of *C. colocynthis* fruits. The antioxidant activity, which is usually attributed to active phytomolecules such as total phenolics, could be affected by the climatic conditions prevailing at different times of the year [7,15].

Polyphenols are natural compounds that exhibit antioxidant and free radical scavenging activities. The antioxidant and free-radical scavenging activities of *C. colocynthis* extracts depend on the presence of phytochemicals, including polyphenols [16]. These phytochemicals act as scavengers of diverse reactive oxygen species such as hydroxyl radicals and peroxyl free radicals [17]. Several studies have assessed the polyphenol content and antioxidant activity in all parts of *C. colocynthis* fruits [17,18,19,20,21]. In addition, other studies have assessed the antioxidant activity in a specific part of the fruit, such as the pulp [6,22], seeds [23,24,25,26], and rind [27]. However, few studies have assessed the antioxidant activity of the different fruit parts. Therefore, the current study is trying to correlate the phenolic contents and antioxidant metabolites to the antioxidant activities of the fruit parts within different accessions at different seasons.

Recently, we reported significant variations in fruit size and morphology, germination requirements (e.g., temperature and light), and genetic variation among 12 accessions within a single *C. colocynthis* population growing in the UAE [28]. In another study, 31 accessions exhibited genetic variations within *C. colocynthis* populations from the Mediterranean coastal region, arid Sinai, and Negev deserts [29]. Interestingly, the genetic diversity in the UAE populations was associated with other fruits’ morphological differences, such as the fruit size, color, stripe pattern, and seed size. For example, the color was dark green in some accessions but light green in others. Furthermore, strips on the rinds were wider and lighter on fruits of some accessions than others. Such variations in morphological, molecular, and germination traits indicate a possible difference in the secondary metabolites and phytomolecules produced in different accessions. Thus, we hypothesize that the variation in fruit and seed colors and sizes could be associated with the production of different levels of phenolic contents and antioxidant activity of different accessions of *C. colocynthis*. Furthermore, the antioxidant activity of *C. colocynthis* extracts collected in different seasons in the UAE has never been investigated. Thus, we hypothesize that the extreme variation in climatic conditions between summer and winter (Supplementary Table S1), especially temperatures, affects the antioxidants activities. Assessing the antioxidant activity of different fruit parts of *C. colocynthis* at different seasons can determine the most suitable fruit part and the best season for using this plant in medication. Besides, screening different accessions could help define the one with the highest antioxidant activity. This accession could be used as a medicinal cash crop with enhanced antioxidant activity for pharmaceutical and industrial uses.

## 2. Materials and Methods

### 2.1. Accession Selection and Growth Conditions

The results from our earlier study indicated significant differences in fruit size, color, and stripe pattern on fruit rinds, temperature and light requirements during the germination stage, and dormancy level of fruits of 12 accessions collected from a single *C. colocynthis* population [28]. The present study used the seeds of three of these accessions that showed the highest genetic variation. Seeds from these accessions (#6, 10, and 13) were soaked in a botanical garden at the University of Sharjah in February 2021. Ten individuals from each accession were grown with about two meters between every two individuals. Plants were irrigated as needed without adding any fertilizers. Plants were treated with a pesticide when we observed an infestation of aphids.

According to the meteorological station at Sharjah Airport, which is the nearest to the experimental site, significant monthly variations in both temperature and humidity were observed. The highest maximum temperatures were recorded in June, July, and August (45–46 °C), while the lowest minimum temperatures were recorded in January and February (7–12 °C).

The humidity in winter and early spring (January–April) was in the range of 94–100%, while in summer (June–September), the humidity was between 79–94% (Supplementary Table S1).

### 2.2. Sample Collection and Preparation

**Figure 1:**
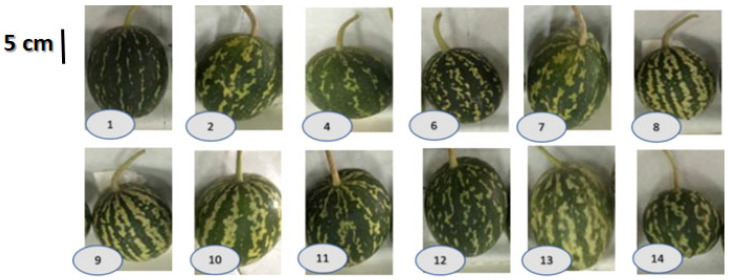
Representative images of fruits of different accessions of one *C. colocynthis* population. The fruits differ in size, color, and rinds’ strip pattern. Fruits of accessions #6, 10, and 13 collected from both summer and winter have been selected in this study.

Fresh green fruits were collected from 5–6 individuals of each accession at the same developmental stage in the summer (July 2020) and winter (January 2021) seasons. Because the fruits of accessions 6, 10, and 13 are different in circumference (22.41 ± 0.6, 29.26 ± 0.6, and 28.05 ± 0.3 cm, respectively), fruit weight (162.0 ± 3.05, 212.2 ± 4.2, and 181.8 ± 3.48 g, respectively), and the weight of 100 seeds (1.90 ± 0.06, 3.07 ± 0.03, and 3.07 ± 0.03 g, respectively), they were selected for this study (Figure 1). The fruits were washed and individually separated into rind, pulp, and seeds.

### 2.3. Sample Extraction

The pulps, rinds, and seeds were dried separately on filter paper in the shade at room temperature (RT) until dryness. After that, they were ground using a lab electric blender into fine powders. Air-dried powder samples (100 mg) of C. colocynthis were extracted by soaking in 1 mL 100% methanol overnight, followed by water bath sonication at RT for 1 h. The extracts were filtered using Whatman No.1 filter paper (Whatman International Ltd., Maidstone, UK). The methanolic extracts were dried using a rotatory evaporator at 45 °C. The residue was dissolved in methanol prior its use for antioxidant analysis. All extracts and analyses were performed in triplicates.

### 2.4. Antioxidant Assays of C. colocynthis

To estimate the antioxidant activity, a method that involved the generation of reactive oxygen species (ROS) was employed [30]. The presence of an antioxidant is determined by the disappearance of ROS and free radicals in a tested sample [30]. The antioxidant activity of *C. colocynthis* methanolic extracts was determined by measuring the total phenolic content (TPC) and DPPH (2,2-diphenyl-1-picryl-hydrazyl-hydrate) free radical scavenging assays.

### 2.5. Total Phenolics Content (TPC) Assay

The quantification of phenolic contents was performed using Folin–Ciocalteu reagent according to [31]. Briefly, 10 µL methanol plant extracts (3 mg/mL) were mixed with 100 µL of 10× diluted Folin–Ciocalteu reagent at final concentrations of 30 µg/mL in 96-well plates. The solution was mixed and incubated in the dark at RT for 5 min. This was followed by adding 90 µL of 7.5% saturated sodium carbonate solution and then incubated at RT in the dark for another 2 h. The absorbance was measured at 750 nm using a microplate reader (Crocodile, Titertek Berthold, Bad Wildbad, Germany). Similarly, gallic acid standards were used to generate a calibration curve at 3, 6, 12.5, 25, 50, 75, 100, 150, 300, and 400 µg/mL. The obtained readings were analyzed using Excel and GraphPad prism 5.0 for windows (GraphPad Software, La Jolla, CA, USA). The phenolic concentration of plant extracts was expressed as gallic acid equivalents (GAE).

### 2.6. DPPH Free Radical Scavenging Assay

The DPPH (2,2-diphenyl-1-picryl-hydrazyl-hydrate) radical scavenging assay was used to assess the free radicals of all *C. colocynthis* fruit parts (rinds, pulps and seeds) according to a protocol adapted from [32] with minor modifications. Briefly, 50 mL DPPH reagent was prepared by dissolving 3.95 mg of DPPH powder in 50 mL of pure methanol to obtain a final concentration of 0.08 mg/mL. The methanolic extracts and the positive control, butylated hydroxytoluene (BHT), were dissolved in methanol and used at different concentrations (1, 10, 50, 100, 250, and 500 µg/mL). DPPH was mixed with methanol and set as a negative control. All extracts and controls were added to a 96-well plate in triplicates. About 50 μL of each extract was mixed with 50 μL of DPPH reagent. The plate was then wrapped in foil and left in the dark for 60 min at RT. The reaction endpoint was detected by the change in the color of the DPPH reagent from dark-violet to light- yellow when it reacted with the antioxidants in the sample [33]. The absorbance was measured quantitatively at 515 nm using a microplate reader (Epoch TM 2 Microplate spectrophotometer, BioTek Instruments, Inc., Winooski, VT, USA). Data were analyzed using Excel and the percentage of free radical scavenging activity was measured as a decrease in the absorbance of DPPH and calculated using the following formula:

Scavenging % of DPPH = [(Abs negative control − Abs sample)/Abs negative control] × 100

### 2.7. Metabolomics Analysis by GC–MS

Metabolites of *C. colocynthis* rind, pulp, and seed methanolic extracts were analyzed using GC–MS according to Semreen et al., 2019 [34] and Altaie et al., 2021 [35]. The methanol in all samples was evaporated, and the dried extracts were derivatized by 150 µL of N-trimethylsilyl-N-methyl trifluoroacetamide, followed by incubation in an oven for 1 h. After that, about 150 µL of hexane was added and then incubated in the oven for another 1 h. The GC–MS analysis was performed using a QP2010 gas chromatograph-mass spectrometer (AOC-20i+s, Shimadzu, Japan) equipped with an autosampler, and using Rtx-5 ms column (30 m length × 0.25 mm inner diameter × 0.25 µm film thickness: Restek, Bellefonte, PA, USA). Helium (99.9% purity) was used as the carrier gas, and the column flow rate was 1.32 mL/min. The initial column temperature was set at 60 °C for 3 min, with 7 °C/min increase to reach 140 °C. Then, the temperature was increased at a rate of 5 °C/min until reaching 300 °C and kept for 5 min. The injection volume was 1 µL, and the injection temperature was 270 °C using a splitless injection mode. Both the ion source temperature and the interface temperature were set at 200 °C and 270 °C, respectively. The MS mode was set on scan mode starting from 50 to 650 *m/z* with a scan speed of 2500. The data were collected and analyzed using MSD Enhanced Chemstation software (Shimadzu, Japan), then exported to Excel for statistical analysis. Product spectra were identified by comparing the measured fragmentation patterns to those found in the NIST-14 Mass Spectral Library.

### 2.8. Statistical Analysis

The data were collected and graphed using Microsoft Excel^®^ and then exported to GraphPad prism 5.0 for windows (GraphPad Software, La Jolla, CA, USA). Three-way ANOVAs were used to assess the main factors (accession, season of collection, and fruit parts) on the free radical scavenging activity (antioxidant activity) and phenolic compound concentration as dependent variables. The data were represented in bar graphs with mean ± standard error (SE) of at least three independent replicas. To analyze the GC–MS data, a heatmap and principal component analysis (PCA) were performed using R [36] with the highly significant metabolites that have antioxidant activity, according to databases including Pubchem, Chebi, and a literature search. The heatmap was done based on Ward’s minimum variance method of clustering and the distances between extract samples were calculated using the Euclidean distance.

## 3. Results

The methanolic extracts of *C. colocynthis* rind, pulp, and seeds collected from three different accessions were screened for their antioxidant activity using TPC and DPPH free radical scavenging assays. The three-way ANOVA indicated significant effects of accessions, seasons, and fruit parts and their interactions on the level of total phenolics and antioxidant activity (*p* < 0.001, Table 1).

### 3.1. Fruits’ Rind Showed the Highest Phenolic Contents Followed by Pulp and Seed

**Figure 2:**
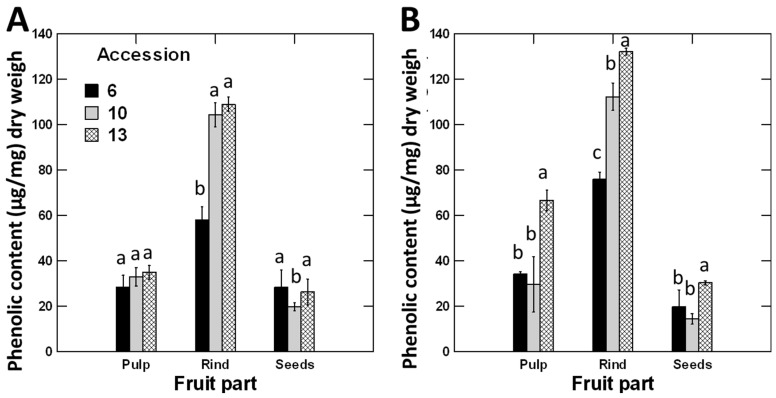
Total phenolic contents of pulps, rinds, and seeds of *C. colocynthis* fruits collected during (**A**) summer and (**B**) winter seasons. Phenolic contents were quantified using Folin–Ciocalteu reagent and expressed in relation to gallic acid equivalents (GAE). Error bars represent the standard error of the mean of three replicates. Within each fruit part, the means of accessions with the same letter (a, b or c) are not significantly different from each other (*p* > 0.05), according to Duncan’s multiple range tests.

The phenolic contents were significantly greater in the rinds by two to fivefold than the pulps and seeds, respectively (Figure 2A). Furthermore, the phenolic contents of the pulps were 1.6 times greater compared to those of the seeds (Figure 2A).

### 3.2. Phenolic Contents of Fruit Parts Were Higher in Winter Than in Summer

There was a significant effect (*p* < 0.001) for the interaction between accession, season, and fruit parts on the phenolic contents, indicating that the phenolic contents of a certain fruit part depend on the time of fruit maturation and the accession (Table 1). For example, the phenolic contents of rinds of accessions 10 and 13 were significantly higher than those of accession 6 in summer. In winter, however, the three accessions differed significantly and could be sorted in the order 13 > 10 > 6 (Figure 2). A similar trend was noticed in pulp samples; no significant difference between the three accessions in summer, but accession 13 attained significantly higher phenolic levels than accessions 6 and 10 in winter samples. The phenolic content was low in seed samples, and there were little variations between the different accessions in both summer and winter (Figure 2).

### 3.3. Free Radical Scavenging Activity Depended on Fruit Parts, Accessions, and Seasons, with Seeds and Summer Showing the Highest Values

**Figure 3:**
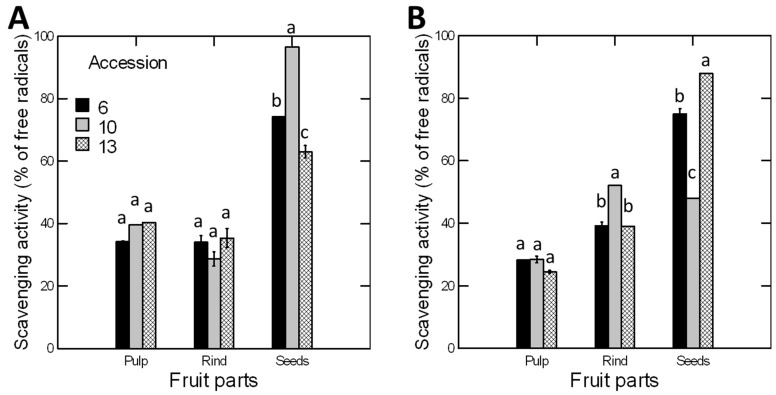
Free radical scavenging activity of pulp, rind, and seeds of three accessions of *C. colocynthis* fruits collected during (**A**) summer and (**B**) winter seasons. Scavenging activity was assessed using a DPPH free radical scavenging assay and the same dry weight of fruit parts. Values were calculated as a relative percentage to the positive control (BHT). Error bars represent the standard error of the mean of three replicates. Within each fruit part, the means of accessions with the same letter (a, b, or c) are not significantly different from each other (*p* > 0.05), according to Duncan’s multiple range tests.

There were significant effects for the season, accession, and fruit parts and all their interactions on the radical scavenging activity of *C. colocynthis* (Table 1). The overall scavenging activity was significantly greater in seeds (74.1% of the free radicals), than in both pulp (32.6%) and rinds (38.0%). Similarly, the overall scavenging activity, regardless of fruit parts, was significantly greater in summer than winter and in accession 10 than accession 6 (Figure 3). The significant interaction between accession, season, and fruit part indicates that the free radical scavenging activity of a certain fruit part depends on the time of fruit maturation and the accession. For example, the scavenging activity in fruit pulp was significantly greater in summer than in winter in all accessions, but the opposite was true for fruit rind; the scavenging activity was greater in winter than in summer. In seeds, the scavenging activity was twice greater in summer than in winter for accession 10 but was 39% greater in winter than in summer for accession 13 (Figure 3).

Together, the phenolic contents and free radicle scavenging activity varied between seasons, accessions, and fruit parts. The highest phenolic contents were observed in the rinds and during the winter. However, the free radicle scavenging activity was higher in the seeds and summer except for accession 13. To confirm the obtained results, the associated antioxidant metabolites were compared via a metabolomics analysis as shown below.

### 3.4. Metabolomics Analysis of C. colocynthis Revealed the Presence of 28 Metabolites with Significant Antioxidant Activities

The metabolites identified from the GC–MS analysis were screened to select only the compounds with reported antioxidant activities using databases such as PubChem and ChEBI associated with a literature search. The screening revealed that 28 metabolites possessed antioxidant activity. The metabolites are listed in Supplementary Table S2. Of the 28 metabolites, 12 molecules were reported only in the summer collection and two only in the winter collection. Among the molecules recorded only in summer, seven were detected only once in a fruit part of a certain accession, three recorded twice, one three times, and one five times. The overall results indicate that the occurrence frequency of antioxidant molecules in the summer was 76 times (23, 27, and 16, in rinds, pulp, and seeds, respectively) and 53 times in winter (21, 22, and 10 in rind, pulp, and seeds, respectively). Among the molecules recorded only once in the summer, four were in the rind of accession 6 (β-D-talopyranose, 1-monolinolein, D-psicopyranose, and ferulic acid), and two in the rinds of accession 10 (β-sitosterol and butanoic acid). In addition, one of the two molecules recorded in the winter (propionic acid) was also present in the rind of accession 6, and the other (1-octacosanol) was present in the rinds of the three accessions in addition to the pulp of accession 10. Such results indicate that the most unique antioxidant metabolites are mainly present in the fruit rinds, especially of accession 6. The results also indicate that the antioxidants frequency was much higher in summer, especially in the rinds, than in winter. This result agrees with the total phenolic content results that the highest total phenolics were in the rinds of the three accessions (Figure 2).

The selected metabolites were further categorized by a heatmap and principal component analysis (PCA).

### 3.5. Antioxidant’s Metabolites Were Clustered Based on Fruit Parts

**Figure 4:**
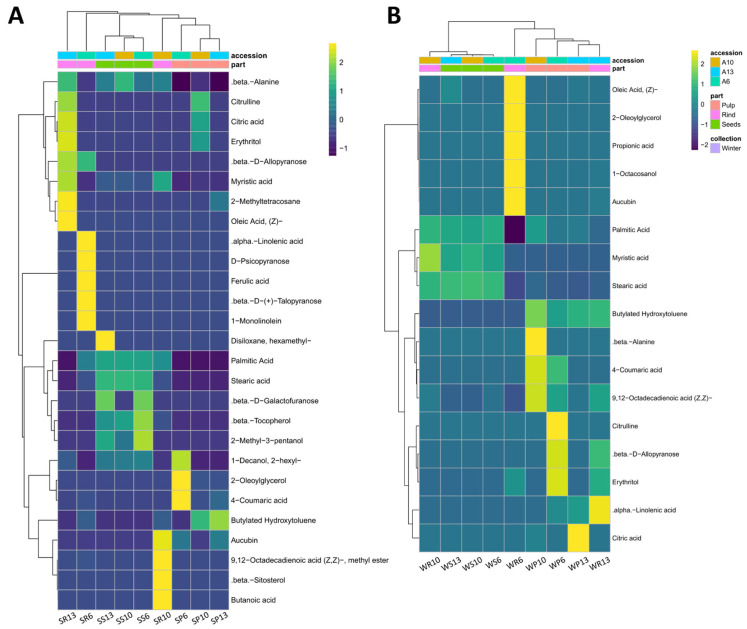
Heatmap of the most significant antioxidant metabolites of *C. colocynthis*. The color range represent the standardized values of the average relative percentages of the metabolites. The comparisons are performed based on the different seasons, including (**A**) summer and (**B**) winter. The first letter in the sample name indicates the collection season (S: summer, W: winter), followed by a letter indicating the analyzed fruit part (P: pulp, S: seed, R: rind), and then by the accession number (6, 10, 13).

A heatmap analysis clustered the antioxidant metabolites based on the accessions, the fruit parts, and the seasons (Figure 4). The total number of metabolites that appeared in summer (27 compounds) was larger than in winter (17 compounds). A metabolites comparison based on the season revealed that metabolites were clustered based on fruit’s part. Such clustering was more obvious in the summer collection than in the winter collection. Rind samples did not cluster together, while the pulp and seed samples from the different accessions were clustered within the collection seasons. Metabolites from rind samples were partially clustered in the summer (Figure 4A) and completely separated in the winter (Figure 4B). Some antioxidant compounds were restricted to specific organs at a specific season. For example, 2-methyl-3-pentanol existed only in the seeds of the summer collection, while 9,12-octadecadienoic acid existed in the rinds (Supplementary Table S2).

Some metabolites appeared to be prominent in both summer and winter (Figure 4A,B). Those include saturated fatty acids such as palmitic acid and stearic acid. In the winter, both fatty acids attained high concentrations in all fruit parts of the three accessions, except for the rind of accession 6. Interestingly, palmitic acid and stearic acid constituted 99.3–99.7% of the total antioxidant activity of seeds of the winter and 89.6–91.6% of the summer collections of the three accessions. Moreover, these two fatty acids were also present at moderate-to-high concentrations in pulps and rinds of different accessions of the winter collection but only in the rinds of the summer collection. In the summer collection, 2-oleoylglycerol represented 86.4% of the total antioxidant compounds in the pulps of accession 6 but attained low concentrations in other parts of all accessions. Furthermore, 2-hexyl-1-decanol, butylated hydroxytoluene, and citric acid were recorded on many occasions in both summer and winter collections.

The numbers and concentrations of the antioxidant metabolites varied greatly among the different accessions. β-D-allopyranose, erythritol, and oleic acid were the most dominant in the rind sample of accession 13, and 2-hexyl-1-decanol was the most dominant in the rind sample of accessions 10 and 13. In the pulp sample, 4-coumaric acid was the most dominant in accessions 6 and 10, while butylated hydroxytoluene was dominant in accessions 10 and 13. Moreover, aucubin was found in high concentration in the rinds of the winter and summer samples of accessions 6 (59% of the total antioxidant molecules) and 13 (11.3%) (Supplementary Table S2).

Interestingly, some of the metabolites were presented in more than one accession only in the summer samples (e.g., α-linolenic acid, β-tocopherol, 2-methyl-3-pentanol, and 9, 12-octadecadienoic acid) (Figure 4A), or winter samples (e.g., 1-octacosanol) (Figure 4B).

### 3.6. Principal Component Analysis (PCA)

**Figure 5:**
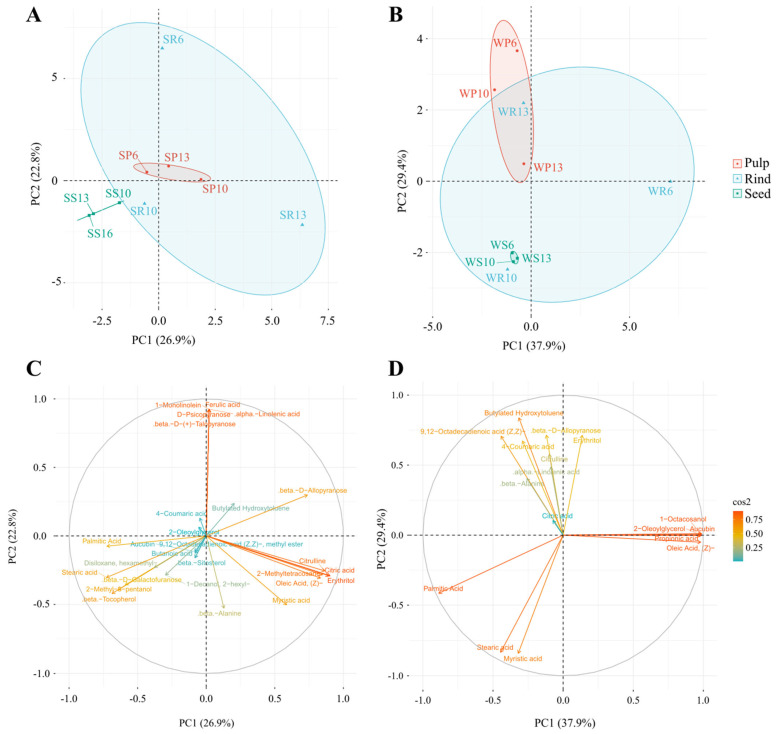
Principal component analysis (PCA) of the selected antioxidant metabolites detected in *C. colocynthis* pulp, rind, and seed samples. This figure shows PCA individual’s plot of samples collected in (**A**) summer and (**B**) winter seasons with the corresponding loadings plots of (**C**) summer and (**D**) winter collected samples. The first letter in the sample name indicates the collection season (S: summer, W: winter), followed by a letter representing the analyzed fruit part (P: pulp, S: seed, R: rind), and then by the accession number (6, 10, 13).

To gain more insight on the effects of season, fruit part, and accession on the presence and amount of antioxidant compounds, a PCA was performed. Based on the season, the three accessions of *C. colocynthis* rind, pulp, and seeds samples were separated in summer (Figure 5A) and winter (Figure 5B). Summer samples were separated by the first principal component (PC1) and the second principal component (PC2), representing 26.9% and 22.8% of the total variation, respectively. On the other hand, winter samples represented 37.9% and 29.4% of the total variation, respectively. Such results indicated that the variation in the studied antioxidant could be explained more with the accession and fruit parts in winter than in summer. The variation in metabolites levels was noticed in the rind samples, particularly in the winter season (Figure 5B). The overall PCA analysis of the 28 metabolites indicated a good separation of seeds and pulp samples in both summer and winter seasons (Figure 5A,B).

The loadings plot results of the metabolites detected in seeds collected in summer (Figure 5C) were similar to those in the heatmap (Figure 4A). The plots resulted in three main clusters. The first was for the antioxidants present only in the seeds of at least one accession during the summer (e.g., 2-methyl-3-pentanol, β-tocopherol, and β-D-galactofuranose) or in all parts of the fruits but with the highest concentrations in the seeds (e.g., palmitic acid and stearic acid). The second cluster was for the antioxidants present in the rind of accession 6 in the summer (e.g., α-linolenic acid, β-D-(+)-talopyranose, 1-monolinolein, D-psicopyranose, and ferulic acid). The third cluster was for the antioxidants present in the rind and pulp, but rarely in seeds (e.g., citric acid, citrulline, 2-methyltetracosane, erythritol, myristic acid, and oleic acid, (Z)-). Interestingly, β-D-allopyranose was separated alone, which presented at high concentrations in the rinds of accessions 6 and 13 (Figure 5C).

The loadings plot results of the metabolites detected in the winter (Figure 5D) were similar to those in the heatmap (Figure 4B). For example, one cluster contained the antioxidants that existed in the different fruit parts of the different accessions with high concentrations (e.g., palmitic acid and stearic acid) or low concentrations (e.g., myristic acid). In addition, another group of antioxidants presented in the pulps and/or rinds of some accessions (e.g., β-D-allopyranose, erythritol, and 4-coumaric acid) (Figure 5D).

**Figure 6:**
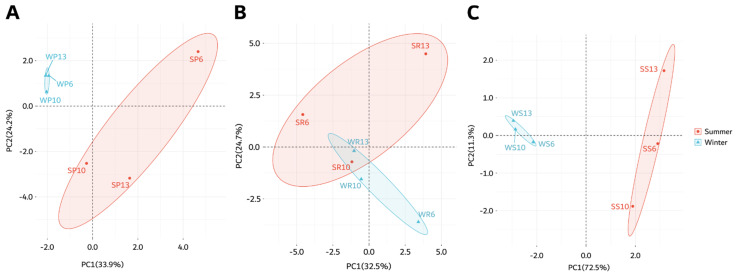
Principal component analysis (PCA) of the selected antioxidant metabolites detected in (**A**) pulp, (**B**) rind, and (**C**) seed samples collected in summer and winter seasons. The scores are plotted with confidence intervals based on the collection season. The first letter in the sample name indicates the collection season (S: summer, W: winter), followed by a letter representing the analyzed fruit part (P: pulp, S: seed, R: rind), and then by the accession number (6, 10, 13).

The metabolites of the pulp showed a good separation between the summer and winter collections (Figure 6A), while the rind samples did not separate based on the collection season (Figure 6B). This was consistent with the results from the heatmap represented in Figure 4. Additionally, there was a good separation between the summer and winter seeds samples (Figure 6C), consistent with the heatmap in Figure 4.

## 4. Discussion

The present study assessed the phenolic contents, antioxidants activities, and antioxidant profile of *C. colocynthis*’ different fruit parts (rind, pulp, and seeds) from genetically different accessions collected in the summer and winter seasons. The results showed significant effects for the season of fruit collection, accession, fruit parts, and all their interactions on the phenolic content and DPPH free radical scavenging ability. The overall results indicated that the response of phenolics and antioxidants in different fruit parts depended on the prevailing environmental conditions and genetics of the individuals. According to the authors’ knowledge, it is the first time phenolics and antioxidants in *C. colocynthis* have been assessed in such a design. This could help define the proper season, fruit part, and accession to maximize the fruits’ antioxidant activities.

### 4.1. Phenolic Contents and Antioxidants Responded Differently to the Season

Plants produce several secondary metabolites, including flavonoids and phenolic acids, to protect them from biotic (e.g., herbivores, pathogens) and abiotic stresses (e.g., temperature, drought, and salinity). These compounds have a high antioxidant activity to scavenge reactive oxygen species produced under environmental stresses. Therefore, the phenolic contents with antioxidant compounds depend on environmental factors [37]. The overall average of the phenolic content of the winter fruits was significantly greater than summer, but the reverse was true for the overall antioxidant; it was greater in summer than winter (Figure 2 and Figure 3). The high phenolic contents in the outer parts (e.g., rinds) could protect the fruits from the high levels of ROS that can induce oxidative damage under extreme temperatures, i.e., low minimum winter temperatures (7 °C in January) and maximum summer temperature (up to 45–46 °C in May to August) (Supplementary Table S1). Other studies also reported that plants growing at different times of the year might have significant differences in their chemical constituents [9,10]. For example, the contents of main tea flavanols and polyphenols were significantly higher in the warm summer months than in cooler months. This was attributed to the higher temperature, higher light intensity, and longer day length during the summer times [38]. Therefore, seasonal variation in phenolics should be associated with antioxidant activity. Several authors have reported significant effects of climatic conditions at different seasons on antioxidant activity, which is usually attributed to active phytomolecules such as phenolics [7,15,39]. For example, broccoli inflorescences (*Brassica oleracea*) and Portuguese kale (*Brassica oleracea* L. var. *acephala* D.C) showed a greater antioxidant activity in the summer season. In comparison, turnip leaves (*Brassica rapa* L. var. rapa) showed a higher activity in the winter [7]. Moreover, the highest total polyphenol contents and antioxidant activity of *Secondatia floribunda* were reported in the dry season, while the lowest was in the rainy season [15].

### 4.2. Phenolic Compounds Are Rich in Rinds

Polyphenols play crucial roles in plant growth, development, and stress protection [40]. Our results showed a significantly greater TPC in the rind than in the pulp and seeds. The phenolic compounds in the rind were 2.6 and 4.3 times those present in the pulp and seeds, respectively (Figure 2). It has been reported that the outer fruits’ layers, such as rind, peel, and shell, are rich with polyphenolic compounds to protect the inner components, especially the newly developed sensitive seeds [41]. Similar results were reported in four species of cucurbits, where the polyphenolic contents were higher in the peels than the pulps of pumpkin, ash gourd, watermelon, and muskmelon [42]. In addition, in *Sechum edule*, another cucurbit, the peel had higher total phenol, flavonoid, carotenoid contents, and antioxidant activity than the pulp and leaves [43]. It has been reported that the biosynthetic pathway of phenolic compounds is usually activated under environmental stresses [40]. Therefore, phenolic compounds play an important role in protecting plant organs exposed to direct stress by scavenging the free radicals produced in the cells or stopping the oxidation of biomolecules [44]. For example, the peels of pomegranate fruits grown under harsh climatic conditions exhibited a higher antioxidant activity and a higher total phenolics content than those in the peels of fruit grown in more favorable conditions [45]. Other studies have reported that the metabolite accumulation in *C. colocynthis* rinds changes in variable environmental conditions. For example, it has been indicated that the aqueous extract of *C. colocynthis* fruits’ rind was richer in saponin and glycosidic components [27]. However, lower glycoside quantities were reported in the rind than in the pulp, seeds, stems, and leaves [46]. One possible reason for the difference in the glycosidic components between the two studies could be the prevailing environmental condition during fruit development since Abdel-Hassan et al., 2000, collected their samples in autumn (November) from an arid Iraqi population [27]. In contrast, Darwish–Sayed, Balbaa et al., 1974, collected the fruits during winter from the Mediterranean climate of Egypt [43]. Such a result indicates a significant role of environmental conditions in controlling the production of the metabolites in *C. colocynthis* fruit rind.

### 4.3. Antioxidant Activity Is Higher in Seeds

The results of our study indicated that the antioxidant activity of seeds was 2.27 and 1.95 times higher than that of the pulp and rind, respectively. Other studies supported a high antioxidant activity in seeds of *C. colocynthis.* For example, Gill, Kaur et al., 2011, showed that the seed extract of *C. colocynthis* exhibited a maximum percentage inhibition of 79.4 and 72.4% by DPPH and hydrogen peroxide methods, respectively, indicating a good antioxidant potential [24]. In addition, the total phenolic and flavonoid compounds of the crude extract of *C. colocynthis* seeds were 1.33 and 2.41 mg/g dry weight, respectively [47]. Such results indicate the importance of seed protection. Embryos within the seeds are the fruit’s most sensitive and valuable tissue. Plants produce several antioxidants to protect embryos from adverse environmental conditions [48]. The production of antioxidants such as phenols and flavonoids scavenges the ROS produced under adverse conditions [49].

### 4.4. Antioxidant Activity Is Negatively Associated with Phenolic Contents

The present study showed a negative correlation between TPC and the DPPH free radical scavenging ability. Whereas the TPC was significantly greater in the rinds of the three accessions than the seeds (Figure 3), an opposite trend was observed for the DPPH free radical scavenging ability. The DPPH free radical scavenging ability was significantly greater in seeds than in rinds and pulps (Figure 4). Such a result indicates that the level of antioxidant activity depends on the type and levels of the antioxidant compounds rather than the TPC. It also indicates that the phenolic compounds are not the sole compounds responsible for antioxidant activity. In fact, the antioxidant activity results from the combined effects and the cooperation between several metabolites, including organic acids, polyphenols, carotenoids, vitamins, and peptides [50]. In our study, the PCA loadings plot of the antioxidant metabolites of *C. colocynthis* detected a cluster with seven antioxidants occurring only in the seeds of at least one accession during the summer. These compounds were 2-methyl-3-pentanol, β-D-galactofuranose, and β-tocopherol. Additionally, three important antioxidant fatty acids (oleic acid, palmitic acid, and stearic acid) were recorded with significantly higher concentrations in the seeds than in the rinds and pulp. The average relative concentrations of oleic acid, palmitic acid, and stearic acid in the two seasons and the three accessions were 17.2, 44.49, and 33.52%, respectively, of the total antioxidant activity in the seeds, compared to 9.1, 13.92, and 12.41% in the rind, and 0.2, 13.0, and 5.84% in the pulp (Supplementary Table S2). These three fatty acids were the main components that exhibited a significant antioxidant activity in the seed oils of three cucurbits (*Lagenaria siceraria*, *Lagenaria breviflora*, and *Luffa cylindrica* [51]), *Phoenix dactylifera*, *P. theophrasti* [50], and six almond varieties [52]. The overall results indicate that the antioxidant activity is higher in the fatty acids than other phenolic and flavonoid compounds.

### 4.5. Accession Affects the Phenolic Content and Antioxidant Activity

Our results showed significant variations in both phenolic contents and DPPH free radical scavenging ability among the different accessions. Such a variation was affected by season and fruit parts. For example, the phenolic content in summer was significantly greater in all parts of accession 13 fruits than in those of accessions 6 and 10. However, there was no significant difference in the phenolic content of seeds and pulp of the different accessions in winter. The rinds of accession 6 had the lowest phenolic content. Such results may indicate that the difference in phenolic contents and antioxidant activities could be attributed to the intensity of the rinds’ green color of different fruits accessions. Furthermore, there is an inverse relationship between the green color and strips of the rinds (Figure 1). The light green color with wider strips of accession 13 rinds could be associated with a higher phenolic content, but the dense green color and narrower strips of accession 6 were associated with a lower phenolic content. In other plants, such as *Capsicum annuum*, the environmental stresses stimulated by UV radiation decreased the pigment concentration but increased the antioxidant activity [53]. A similar result was reported in *Sorghum vulgare* [54].

The results also showed a significant variation in seed antioxidant activity among the different accessions. However, there was no significant difference in rind and pulp antioxidant activities between the different accessions. In seeds, the antioxidant activity was significantly greater in accession 10 of the summer collection and in accession 13 of the winter collection (Figure 3). The higher antioxidant activity in summer and winter seeds of accessions 10 and 13 cannot be explained by the level of total phenolic compounds but by the type of dominant antioxidant compounds. For example, palmitic acid and stearic acid constitute approximately 99.3% of the total antioxidant compounds in the seeds of accession 10. Both fatty acids showed very strong potent antioxidant activity in vitro and in mouse model [55]. These fatty acids exert a high antioxidant activity in the seed oils of other plant species [50,51,52].

### 4.6. Future Perspectives

In *C. colocynthis*, the phenolic and antioxidant compounds depend on environmental conditions associated with the seasons, fruit parts, and accession. Therefore, it is important to assess the impacts of environmental conditions associated with the seasonal variation on the yield and biological activities of the phenolic and antioxidant compounds in different parts of this important medicinal plant. There are extreme variations in the environmental conditions in the UAE. For example, temperatures vary from 45–46 °C during the summer to only 7 °C during the winter (Supplementary Table S1). In addition, some years receive an extremely high amount of rainfall (above 300 mm), but several other continuous years receive less than 10–15 mm of rainfalls [56]. Therefore, under controlled conditions, it is recommended to assess the impacts of individual environmental conditions associated with the yearly and seasonal variations on the yield and biological activities of biomolecules in *C. colocynthis*. This could help define the most suitable range in each of different environmental conditions for the commercial production of each medicinally important biomolecule [13]. Furthermore, the quality and quantity of phytochemicals should be measured upon the duration and frequency of different biotic and abiotic stresses such as drought, light, salinity, temperature, etc. [37,57].

## 5. Conclusions

The three accessions of *C. colocynthis* fruits used showed variable phenolic contents and antioxidant activities based on fruit collection time and fruit part. This indicates that the biomolecules formed in this species are genetically and environmentally dependent. The results showed that the total phenols were significantly greater in the rinds than in the seeds and pulp, but the antioxidant activity was significantly higher in the seeds than in the rinds and pulps. Despite the presence of 28 metabolites with antioxidant activities, two fatty acids (palmitic acid and stearic acid) may be responsible for the higher antioxidant activity of the seeds. In addition, the higher antioxidant activity in the summer than winter fruits suggests consuming seeds of *C. colocynthis* produced in summer as high-quality antioxidants. The significant variation in the antioxidant activity in different genetic accessions of *C. colocynthis* suggests that this species is a proper genetic resource for enhancing the antioxidant activity for large-scale production.

## Figures and Tables

**Table 1:** Three-way ANOVA assessing the effects of accessions, season of fruit production and fruit parts on (a) phenolic content and (b) scavenging activity of *C. colocynthis* in a population of the hyperarid environment of the UAE. Ns: insignificant difference at *p* ≤ 0.05.

Source of Variation	df	Mean Squares	F-Ratio	*p*
(a) Phenolic content				
Accession	2	3007	63.0	<0.001
Season	1	898.0	18.8	<0.001
Fruit parts	2	28,865	605.2	<0.001
A*S	2	475.2	9.96	<0.001
A*FP	4	1338.7	28.07	<0.001
S*FP	2	470.2	9.85	<0.001
Error	36	47.7		
(b) Antioxidant activity				
Accession	2	8.96	3.30	<0.05
Season	1	93.4	34.4	<0.001
Fruit parts	2	9159.1	3370.7	<0.001
A*S	2	325.8	119.9	<0.001
A*FP	4	21.8	8.03	<0.001
S*FP	2	618.8	227.7	<0.001
Error	36			

## Data Availability

The data are contained within the article and Supplementary Materials.

## Supplementary Materials

The following supporting information can be downloaded at: https://www.mdpi.com/article/10.3390/antiox11040656/s1. Table S1: Monthly variation in temperature and atmospheric humidity in Sharjah Airport, a few Kilometers away from the study site. Table S2: The relative % of metabolites detected in *C. colocynthis* fruits collected in Summer (Red Bold) and Winter (Blue) seasons, from the GC-MS profile results with an antioxidant activity. The analyzed part (R: rind, P: pulp, S: seed). The accession number (6, 10, 13).

## References

[1] Lira-Saade R., Montes-Hernández S., Hernández-Bermejo J.E., León J. (1992). Cucúrbitas (*Cucurbita* spp.). Producción y Protección Vegetal.

[2] Christenhusz M., Byng J. (2016). The number of known plants species in the world and its annual increase. Phytotaxa.

[3] Zaini N.A.M., Anwar F., Hamid A.A., Saari N. (2011). Kundur [*Benincasa hispida* (Thunb.) Cogn.]: A potential source for valuable nutrients and functional foods. Food Res. Int..

[4] El-Keblawy A., Shabana H.A., Navarro T., Soliman S. (2017). Effect of maturation time on dormancy and germination of *Citrullus colocynthis* (Cucurbitaceae) seeds from the Arabian hyper-arid deserts. BMC Plant Biol..

[5] Hussain A.I., Rathore H.A., Sattar M.Z., Chatha S.A., Sarker S.D., Gilani A.H. (2014). *Citrullus colocynthis* (L.) Schrad (bitter apple fruit): A review of its phytochemistry, pharmacology, traditional uses and nutritional potential. J. Ethnopharmacol..

[6] Wasfi I., Bashir A., Abdalla A., Banna N., Tanira M. (1995). Antiinflammatory activity of some medicinal plants of the United Arab Emirates. Int. J. Pharmacogn..

[7] Aires A., Fernandes C., Carvalho R., Bennett R.N., Saavedra M.J., Rosa E.A. (2011). Seasonal effects on bioactive compounds and antioxidant capacity of six economically important Brassica vegetables. Molecules.

[8] Sartor T., Xavier V., Falcão M., Mondin C., Dos Santos M., Cassel E., Astarita L., Santarém E. (2013). Seasonal changes in phenolic compounds and in the biological activities of *Baccharis dentata* (Vell.) GM Barroso. Ind. Crops Prod..

[9] Deng N., Chang E., Li M., Ji J., Yao X., Bartish I.V., Liu J., Ma J., Chen L., Jiang Z. (2016). Transcriptome characterization of *Gnetum parvifolium* reveals candidate genes involved in important secondary metabolic pathways of flavonoids and stilbenoids. Front. Plant Sci..

[10] Lemos M.F., Lemos M.F., Pacheco H.P., Guimarães A.C., Fronza M., Endringer D.C., Scherer R. (2017). Seasonal variation affects the composition and antibacterial and antioxidant activities of *Thymus vulgaris*. Ind. Crops Prod..

[11] Lemos M.F., Lemos M.F., Pacheco H.P., Endringer D.C., Scherer R. (2015). Seasonality modifies rosemary’s composition and biological activity. Ind. Crops Prod..

[12] Sarrou E., Martens S., Chatzopoulou P. (2016). Metabolite profiling and antioxidative activity of Sage (*Salvia fruticosa* Mill.) under the influence of genotype and harvesting period. Ind. Crops Prod..

[13] Sehlakgwe P.F., Lall N., Prinsloo G. (2020). 1H-NMR metabolomics and LC-MS analysis to determine seasonal variation in a cosmeceutical plant *Leucosidea sericea*. Front. Pharmacol..

[14] Trivino M.G., Johnson C.B. (2000). Season has a major effect on the essential oil yield response to nutrient supply in *Origanum majorana*. J. Hortic. Sci. Biotechnol..

[15] Ribeiro D.A., Camilo C.J., Nonato C.d.F.A., Rodrigues F.F.G., Menezes I.R.A., Ribeiro-Filho J., Xiao J., de Almeida Souza M.M., da Costa J.G.M. (2020). Influence of seasonal variation on phenolic content and in vitro antioxidant activity of *Secondatia floribunda* A. DC. (Apocynaceae). Food Chem..

[16] Sebbagh N., Cruciani-Guglielmacci C., Ouali F., Berthault M.-F., Rouch C., Sari D.C., Magnan C. (2009). Comparative effects of *Citrullus colocynthis*, sunflower and olive oil-enriched diet in streptozotocin-induced diabetes in rats. Diabetes Metab..

[17] Hussain A.I., Rathore H.A., Sattar M.Z., Chatha S.A., ud din Ahmad F., Ahmad A., Johns E.J. (2013). Phenolic profile and antioxidant activity of various extracts from *Citrullus colocynthis* (L.) from the *Pakistani flora*. Ind. Crops Prod..

[18] Nmila R., Gross R., Rchid H., Roye M., Manteghetti M., Petit P., Tijane M., Ribes G., Sauvaire Y. (2000). Insulinotropic effect of *Citrullus colocynthis* fruit extracts. Planta Med..

[19] Kumar S., Kumar D., Saroha K., Singh N., Vashishta B. (2008). Antioxidant and free radical scavenging potential of *Citrullus colocynthis* (L.) Schrad. methanolic fruit extract. Acta Pharm..

[20] Hsouna A.B., Alayed A.S. (2012). Gas chromatography-mass spectrometry (GC-MS) analysis and in vitro evaluation of antioxidant and antimicrobial activities of various solvent extracts from *Citrullus colocynthis* (L.) roots to control pathogen and spoilage bacteria. Afr. J. Biotechnol..

[21] Rizvi T.S., Mabood F., Ali L., Al-Broumi M., Al Rabani H.K., Hussain J., Jabeen F., Manzoor S., Al-Harrasi A. (2018). Application of NIR spectroscopy coupled with PLS regression for quantification of total polyphenol contents from the fruit and aerial parts of *Citrullus colocynthis*. Phytochem. Anal..

[22] Delazar A., Gibbons S., Kosari A.R., Nazemiyeh H., Modarresi M., Nahar L., Sarker S.D. (2006). Flavone C-glycosides and cucurbitacin glycosides from *Citrullus colocynthis*. Daru.

[23] Jeyanthi K., Christy A. (2009). Antihyperglycemic Effect of *Citrullus colocynthis* Seed Powder in Alloxan-Induced Diabetic Rats. IUP J. Biotechnol..

[24] Gill N., Kaur S., Arora R., Bali M. (2011). Screening of antioxidant and antiulcer potential of *Citrullus colocynthis* methanolic seed extract. Res. J. Phytochem..

[25] Benariba N., Djaziri R., Bellakhdar W., Belkacem N., Kadiata M., Malaisse W.J., Sener A. (2013). Phytochemical screening and free radical scavenging activity of *Citrullus colocynthis* seeds extracts. Asian Pac. J. Trop. Biomed..

[26] Salama H.M., Al Rabiah H.K. (2015). Physiological effects of allelopathic activity of *Citrullus colocynthis* on *Vicia faba* and *Hordeum vulgare*. Eur. J. Biol. Res..

[27] Abdel-Hassan I.A., Abdel-Barry J.A., Mohammeda S.T. (2000). The hypoglycaemic and antihyperglycaemic effect of *Citrullus colocynthis* fruit aqueous extract in normal and alloxan diabetic rabbits. J. Ethnopharmacol..

[28] Alnablsi S., El-Keblawy A., Mosa K.A., Soliman S. (2021). Variation among individuals of *Citrullus colocynthis* from a desert population in morphological, genetic, and germination attributes. Trop. Ecol..

[29] Zamir D., Navot N., Rudich J. (1984). Enzyme polymorphism in Citrullus lanatus and *C. colocynthis* in Israel and Sinai. Plant Syst. Evol..

[30] Cao G., Alessio H.M., Cutler R.G. (1993). Oxygen-radical absorbance capacity assay for antioxidants. Free. Radic. Biol. Med..

[31] Sánchez-Rangel J.C., Benavides J., Heredia J.B., Cisneros-Zevallos L., Jacobo-Velázquez D.A. (2013). The Folin–Ciocalteu assay revisited: Improvement of its specificity for total phenolic content determination. Anal. Methods.

[32] Koleva I.I., Van Beek T.A., Linssen J.P., Groot A.d., Evstatieva L.N. (2002). Screening of plant extracts for antioxidant activity: A comparative study on three testing methods. Phytochem. Anal. Int. J. Plant Chem. Biochem. Tech..

[33] Sudhanshu N.R., Mittal S., Menghani E. (2012). Antioxidant activity of *Solanum surratense* and *Solanum nigrum* methanolic extract: An in vitro evaluation. JAPR.

[34] Semreen M.H., Soliman S.S.M., Saeed B.Q., Alqarihi A., Uppuluri P., Ibrahim A.S. (2019). Metabolic Profiling of Candida auris, a Newly-Emerging Multi-Drug Resistant Candida Species, by GC-MS. Molecules.

[35] Altaie A.M., Venkatachalam T., Samaranayake L.P., Soliman S.S.M., Hamoudi R. (2021). Comparative Metabolomics Reveals the Microenvironment of Common T-Helper Cells and Differential Immune Cells Linked to Unique Periapical Lesions. Front. Immunol..

[36] Team R.C. (2013). R: A Language and Environment for Statistical Computing.

[37] Mahajan M., Kuiry R., Pal P.K. (2020). Understanding the consequence of environmental stress for accumulation of secondary metabolites in medicinal and aromatic plants. J. Appl. Res. Med. Aromat. Plants.

[38] Yao L., Caffin N., D’arcy B., Jiang Y., Shi J., Singanusong R., Liu X., Datta N., Kakuda Y., Xu Y. (2005). Seasonal variations of phenolic compounds in Australia-grown tea (*Camellia sinensis*). J. Agric. Food Chem..

[39] Abouleish M., El-Keblawy A., Mosa K.A., Soliman S.S.M. (2020). Importance of Environmental Factors on Production of Computationally-Defined Natural Molecules against COVID-19 Pandemic. Curr. Top. Med. Chem..

[40] Šamec D., Karalija E., Šola I., Vujčić Bok V., Salopek-Sondi B. (2021). The Role of polyphenols in abiotic stress response: The influence of molecular structure. Plants.

[41] Jeong S.-M., Kim S.-Y., Kim D.-R., Jo S.-C., Nam K., Ahn D., Lee S.-C. (2004). Effect of heat treatment on the antioxidant activity of extracts from citrus peels. J. Agric. Food Chem..

[42] Singh J., Singh V., Shukla S., Rai A. (2016). Phenolic content and antioxidant capacity of selected cucurbit fruits extracted with different solvents. J. Nutr. Food Sci..

[43] Loizzo M.R., Bonesi M., Menichini F., Tenuta M.C., Leporini M., Tundis R. (2016). Antioxidant and carbohydrate-hydrolysing enzymes potential of *Sechium edule* (Jacq.) Swartz (Cucurbitaceae) peel, leaves and pulp fresh and processed. Plant Foods Hum. Nutr..

[44] Skandrani I., Limem I., Neffati A., Boubaker J., Sghaier M.B., Bhouri W., Bouhlel I., Kilani S., Ghedira K., Chekir-Ghedira L. (2010). Assessment of phenolic content, free-radical-scavenging capacity genotoxic and anti-genotoxic effect of aqueous extract prepared from *Moricandia arvensis* leaves. Food Chem. Toxicol..

[45] Schwartz E., Tzulker R., Glazer I., Bar-Ya’akov I., Wiesman Z., Tripler E., Bar-Ilan I., Fromm H., Borochov-Neori H., Holland D. (2009). Environmental conditions affect the color, taste, and antioxidant capacity of 11 pomegranate accessions’ fruits. J. Agric. Food Chem..

[46] Darwish–Sayed M., Balbaa S., Afifi M. (1974). The glycosidal content of the different organs of *Citrullus colocynthis*. Planta Med..

[47] Nehdi I.A., Sbihi H., Tan C.P., Al-Resayes S.I. (2013). Evaluation and characterisation of *Citrullus colocynthis* (L.) Schrad seed oil: Comparison with *Helianthus annuus* (sunflower) seed oil. Food Chem..

[48] Bailly C. (2004). Active oxygen species and antioxidants in seed biology. Seed Sci. Res..

[49] Sharma A., Shahzad B., Rehman A., Bhardwaj R., Landi M., Zheng B. (2019). Response of phenylpropanoid pathway and the role of polyphenols in plants under abiotic stress. Molecules.

[50] Liolios C., Sotiroudis G., Chinou I. (2009). Fatty acids, sterols, phenols and antioxidant activity of Phoenix theophrasti fruits growing in Crete, Greece. Plant Foods Hum. Nutr..

[51] EE E., II U. (2013). Physicochemical properties, fatty acids composition and antioxidant activity of some cucurbits seed oils. IJBPAS.

[52] Csakvari A.C., Lupitu A., Bungău S., Gitea M.A., Gitea D., Ţiţ D.M., Copolovici L., Nemeth S., Copolovici D. (2019). Fatty acids profile and antioxidant activity of almond oils obtained from six *Romanian varieties*. Farmacia.

[53] Mahdavian K., Ghorbanli M., Kalantari K.M. (2008). The effects of ultraviolet radiation on the contents of chlorophyll, flavonoid, anthocyanin and proline in *Capsicum annuum* L.. Turk. J. Bot..

[54] Ambasht N.K., Agrawal M. (1998). Physiological and biochemical responses of Sorghum vulgare plants to supplemental ultraviolet-B radiation. Can. J. Bot..

[55] Cho K.-H., Hong J.-H., Lee K.-T. (2010). Monoacylglycerol (MAG)-oleic acid has stronger antioxidant, anti-atherosclerotic, and protein glycation inhibitory activities than MAG-palmitic acid. J. Med. Food.

[56] Feulner G.R. (2006). Rainfall and climate records from Sharjah Airport: Historical data for the study of recent climatic periodicity in the UAE. Tribulus.

[57] Mahendra C., Chandra M.N., Murali M., Abhilash M., Singh S.B., Satish S., Sudarshana M. (2020). Phyto-fabricated ZnO nanoparticles from *Canthium dicoccum* (L.) for Antimicrobial, Anti-tuberculosis and Antioxidant activity. Process Biochem..

