# Supplementary material for: Phenolic Contents and Antioxidant Activity of Citrullus colocynthis Fruits, Growing in the Hot Arid Desert of the UAE, Influenced by the Fruit Parts, Accessions, and Seasons of Fruit Collection
Source: Antioxidants (Basel). 2022 Mar 29;11(4):656. doi: 10.3390/antiox11040656 (PMC9031273; doi:10.3390/antiox11040656)
Supplement: Supplementary file 1 [file antioxidants-11-00656-s001.zip › antioxidants-1625021-supplementary.pdf]

Supplementary Table S1

Monthly variation in temperature and atmospheric humidity in Sharjah Airport, a few Kilometers away from the study site.

**Climatic variable**		**Oct. 20**	**Nov. 20**	**Dec. 20**	**Jan. 21**	**Feb. 21**	**Mar. 21**	**Apr. 21**	**May. 21**	**Jun. 21**	**Jul. 21**	**Aug. 21**	**Sep. 21**
**Temperature**	Maximum	42	36	33	28	33	39	41	45	46	45	46	43
**(°C)**	Minimum	18	17	12	7	12	12	17	22	26	28	29	26
	Average	30	27	23	19	21	25	29	32	35	37	36	34
**Humidity (%)**	Maximum	89	88	88	100	100	94	100	89	94	79	89	94
	Minimum	3	21	18	11	12	6	6	3	7	11	6	9
	Average	44	56	54	56	65	47	42	46	47	43	49	54

Supplementary Table S2.

The relative % of metabolites detected in *C. colocynthis* fruits collected in Summer (Red Bold) and Winter (Blue) seasons, from the GC-MS profile results with an antioxidant activity. The analysed part (R: rind, P: pulp, S: seed).The accession number (6, 10, 13).

Compound	Class	References	Summer & Winter								
			A6			A10			A13		
			R	P	S	R	P	S	R	P	S
**α -Linolenic acid**	Fatty acid	(Wang et al., 2007)	**11.21**<br>-	**-**<br>2.4	**-**<br>-	**-**<br>-	**-**<br>-	**-**<br>-	**-**<br>12.18	**-**<br>3.37	**-**<br>-
**β -Alanine**	Amino acid	(Zhao et al., 2016)	**0.19**<br>-	**-**<br>-	**0.41**<br>-	**0.46**<br>-	**0.15**<br>0.23	**0.68**<br>-	**0.7**<br>-	**-**<br>-	**0.44**<br>-
**β -D-(+)-Talopyranose**	Aldohexose sugar	(Chandra et al., 2021)	**0.5**<br>-	**-**<br>-	**-**<br>-	**-**<br>-	**-**<br>-	**-**<br>-	**-**<br>-	**-**<br>-	**-**<br>-
**β -D-Allopyranose**	Aldohexose sugar	(https://www.ebi.ac.uk/chebi/searchId.do?chebiId=CHEBI:40656)	**25.58**<br>-	**-**<br>17.76	**-**<br>-	**-**<br>-	**-**<br>-	**-**<br>-	**36.36**<br>10	**-**<br>-	**-**<br>-
**β -D-Galactofuranose**	polysaccharides	(Hammami et al., 2018)	**-**<br>-	**-**<br>-	**0.14**<br>-	**-**<br>-	**-**<br>-	**-**<br>-	**-**<br>-	**-**<br>-	**0.13**<br>-
**β -Sitosterol**	Phytosterol	(https://www.ebi.ac.uk/chebi/searchId.do?chebiId=CHEBI:27693)	**-**<br>-	**-**<br>-	**-**<br>-	**11.94**<br>-	**-**<br>-	**-**<br>-	**-**<br>-	**-**<br>-	**-**<br>-
**β -Tocopherol**	Fat-soluble vitamin E, methylated phenols	(Burcova et al., 2019)	**0.09**<br>-	**-**<br>-	**3.75**<br>-	**0.62**<br>-	**-**<br>-	**2.29**<br>-	**-**<br>-	**-**<br>-	**2.03**<br>-
**1-Decanol, 2-hexyl-**	fatty alcohol	(Martínez et al., 2009)	**-**<br>2.54	**6.07**<br>-	**2.55**<br>-	**0.52**<br>1.91	**-**<br>0.46	**2.42**<br>-	**1.4**<br>0.03	**-**<br>-	**2.33**<br>-
**1-Monolinolein**	Monoglyceride	(Quijano-Avilés et al., 2021)	**0.95**<br>-	**-**<br>-	**-**<br>-	**-**<br>-	**-**<br>-	**-**<br>-	**-**<br>-	**-**<br>-	**-**<br>-
**1-Octacosanol**	Fatty alcohol	(Sengupta et al., 2018)	**-**<br>24.67	**-**<br>-	**-**<br>-	**-**<br>0.42	**-**<br>0.05	**-**<br>-	**-**<br>0.8	**-**<br>-	**-**<br>-

**2-Methyltetracosane**	Fatty Acyls (Hydrocarbon)	(Ramya et al., 2015)	-<br>-	-<br>-	-<br>-	-<br>-	-<br>-	-<br>-	0.7<br>-	0.17<br>-	-<br>-
**2-Methyl-3-pentanol**	Fatty alcohol	(Ahmed et al., 2019)	-<br>-	-<br>-	0.72<br>-	-<br>-	-<br>-	0.22<br>-	-<br>-	-<br>-	0.44<br>-
**2-Oleoylglycerol**	Monoradylglycerols	(Wang et al., 2014)	-<br>2.54	86.4<br>-	-<br>-	-<br>-	4.11<br>-	-<br>-	-<br>-	2.84<br>-	-<br>-
**4-Coumaric acid**	Phenolic acid / Carboxylic acid	(Ilavenil et al., 2016)	0.25<br>-	4.49<br>0.08	-<br>-	-<br>-	0.22<br>0.15	-<br>-	-<br>-	0.73<br>-	-<br>-
**9,12-Octadecadienoic acid**	Fatty acid methyl ester	(Pinto et al., 2017)	0.18<br>-	-<br>-	-<br>-	4.99<br>-	-<br>-	-<br>-	-<br>-	-<br>-	-<br>-
**Aucubin**	Iridoid o-glycosides	(https://www.medchemexpress.com/Aucubin.html)	-<br>59.33	3.04<br>-	-<br>-	11.32<br>-	-<br>-	-<br>-	-<br>2.29	3.34<br>0.09	-<br>-
**Butanoic acid**	Fatty acid	(Kumar et al., 2010)	-<br>-	-<br>-	-<br>-	0.46<br>-	-<br>-	-<br>-	-<br>-	-<br>-	-<br>-
**Butylated hydroxytoluene**	Methyl phenol	(Iverson 1995)	19.25<br>-	-<br>23.07	-<br>-	18.97<br>-	69.67<br>45.76	-<br>-	-<br>32.35	92.2<br>28.73	-<br>-
**Citric acid**	Organic acid	(Ryan et al., 2019)	-<br>-	-<br>-	0.38<br>-	-<br>0.38	16.42<br>3	0.13<br>-	30.87<br>-	-<br>37.16	0.4<br>-
**Citrulline**	Amino acid	(Ahmadi et al., 2020)	-<br>-	-<br>1	-<br>-	-<br>-	3.57<br>-	-<br>-	4.67<br>-	-<br>-	-<br>-
**D-Psicopyranose**	Hexose	(https://www.ebi.ac.uk/chebi/searchId.do?chebiId=CHEBI:27605)	2.23<br>-	-<br>-	-<br>-	-<br>-	-<br>-	-<br>-	-<br>-	-<br>-	-<br>-
**Disiloxane, hexamethyl-**	Fatty Acyls (Hydrocarbon)	(Otify et al., 2019)	-<br>-	-<br>-	-<br>-	-<br>-	-<br>-	-<br>-	-<br>-	-<br>-	0.13<br>-
**Erythritol**	Sugar alcohol	(Den Hartog et al., 2010)	-<br>5.84	-<br>23.37	-<br>-	-<br>-	4.99<br>-	-<br>-	11.03<br>10.66	-<br>-	-<br>-
**Ferulic acid**	Hydroxycinnamic acid	(Hussain et al., 2013)	0.15	-	-	-	-	-	-	-	-

			-	-	-	-	-	-	-	-	-
**Myristic acid**	Fatty acid	(Gurudeeban et al., 2010)	**0.49**<br>-	**-**<br>-	**2.42**<br>0.35	**7.41**<br>0.71	**0.87**<br>-	**2.62**<br>0.44	**11.7**<br>-	**0.73**<br>-	**2.69**<br>0.38
**Oleic acid**	Fatty acid	(Terés et al., 2008)	**-**<br>2.03	**-**<br>-	**-**<br>-	**-**<br>-	**-**<br>-	**-**<br>-	**2.57**<br>0.03	**-**<br>0.1	**-**<br>0.35
**Palmitic acid**	Fatty acid	(Sawaya et al., 1983)	**24.09**<br>-	**-**<br>22.66	**33.83**<br>33.61	**28.38**<br>34.89	**-**<br>32.62	**34.7**<br>31.21	**-**<br>18.85	**-**<br>22.86	**34.51**<br>32.49
**Propionic acid**	Fatty acid	(Dionisi-Vici et al., 2006)	**-**<br>3.05	**-**<br>-	**-**<br>-	**-**<br>-	**-**<br>-	**-**<br>-	**-**<br>-	**-**<br>-	**-**<br>-
**Stearic acid**	Fatty acid	(Sadou et al., 2007)	**14.83**<br>-	**-**<br>9.66	**55.8**<br>66.04	**14.95**<br>61.68	**-**<br>17.73	**56.93**<br>68.34	**-**<br>12.8	**-**<br>7.68	**56.9**<br>66.77
